# Supplementary material for: Effect of Autotransfusion in HCC Surgery on Survival and Recurrence: A Systematic Review and Meta-Analysis
Source: Cancers (Basel). 2022 Oct 3;14(19):4837. doi: 10.3390/cancers14194837 (PMC9564172; doi:10.3390/cancers14194837)
Supplement: Supplementary file 1 [file cancers-14-04837-s001.zip › cancers-1946485-supplementary.pdf]

Search strategy for Medline via PubMed:

"cell saver" OR "blood salvage" OR autotransfusion OR "auto-transfusion" OR "autologous transfusion") AND (HCC OR "hepatocellular carcinoma" OR hepatocarcinoma OR "primary liver cancer"

"cell saver"[All Fields] OR "blood salvage"[All Fields] OR ("autotransfused"[All Fields] OR "autotransfuser"[All Fields] OR "blood transfusion, autologous"[MeSH Terms] OR ("blood"[All Fields] AND "transfusion"[All Fields] AND "autologous"[All Fields]) OR "autologous blood transfusion"[All Fields] OR "autotransfusion"[All Fields] OR "autotransfusions"[All Fields]) OR "auto-transfusion"[All Fields] OR "autologous transfusion"[All Fields]) AND ("HCC"[All Fields] OR "hepatocellular carcinoma"[All Fields] OR ("carcinoma, hepatocellular"[MeSH Terms] OR ("carcinoma"[All Fields] AND "hepatocellular"[All Fields]) OR "hepatocellular carcinoma"[All Fields] OR "hepatocarcinoma"[All Fields] OR "hepatocarcinomas"[All Fields]) OR "primary liver cancer"[All Fields]
